# Sharing for the common good: Children's levels of social inclusion within the group and their sharing decisions in a public goods game

**DOI:** 10.1111/bjso.70105

**Published:** 2026-06-04

**Authors:** Yael Malin, Hagit Sabato

**Affiliations:** ^1^ Seymour Fox School of Education, The Hebrew University of Jerusalem Jerusalem Israel; ^2^ Max Planck for Human Development Berlin Germany

**Keywords:** cooperation, group dynamics, prosocial behaviour, public goods, sharing decisions, social inclusion

## Abstract

Across two studies, this research explored the relationship between levels of social inclusion within a group and cooperative sharing decisions among children, using the public goods game (PGG). In Study 1 (10–12 years old, *N* = 165), participants' social inclusion was experimentally manipulated using a modified version of the Cyberball paradigm that varied the number of ball tosses, after which they were asked about their willingness to contribute resources to the common pool in the PGG. In Study 2 (10–11 years old, *N* = 249), social inclusion was measured in a naturalistic context by assessing children's interactions with peers in their class and their actual contributions in the PGG at two time points, 1 week apart. In both studies, children who experienced higher levels of social inclusion within the group were more willing to contribute to the group, even at a personal cost, compared with children experiencing lower levels of social inclusion. Together, these findings deepen our understanding of how social inclusion within the group shapes prosocial behaviour during middle childhood—a pivotal period in social development.

The need to belong is a fundamental human motivation, shaping how individuals think, feel and behave in social contexts (Baumeister & Leary, 1995). Empirical research shows that feeling socially connected and supported is positively associated with various prosocial behaviours, such as helping, sharing and cooperating (Angelis & Pensini, 2023; Cowell et al., 2018; Lin et al., 2025; Malin & Sabato, 2026; Pavey et al., 2011; Trombini & Kinias, 2022). Importantly, these links between feeling socially connected and prosocial behaviour may be especially pronounced during middle childhood, a developmental period in which children become increasingly involved in their social environment (Dawes, 2017), and peer relationships become central (Beazidou & Botsoglou, 2016). Understanding these dynamics is important because prosociality plays a key role at both the individual and group levels. Prosocial behaviours are positively associated with children's academic adjustment and psychological well‐being (e.g. Caprara et al., 2000; Eisenberg et al., 2013), and contribute to positive peer relationships and effective group functioning as well (Ma et al., 2020).

As children grow, peers provide a primary context in which social experiences significantly shape self‐perception and interactions with others (Parker et al., 2015). Developmental research has increasingly examined how children's social experiences within the group shape their prosocial tendencies (Stoltz et al., 2016). In particular, previous studies have found a solid positive association between children's social status within the group and their prosociality—in terms of cooperation, helping and kindness towards others (Angelis & Pensini, 2023; Berger et al., 2015; Lin et al., 2025; Warden & MacKinnon, 2003). For example, van den Berg et al. (2015) investigated the association between social status and social behaviour among fifth‐ and sixth‐graders, using peer‐ and teacher‐nomination methods (i.e. children's ratings of classmates' popularity likability and social behaviours; teachers' reports on children's peer status and social behaviours). They found a positive link between popularity and prosocial behaviour, as perceived by peers and teachers, and a negative association between popularity and reported aggressive behaviour. Warden and MacKinnon (2003) examined this link among 9–10‐year‐olds, using children's self‐reports of their social status, and their ratings by their peers. They revealed that the children's social status was positively correlated with self‐reported prosocial behaviours (inclusion, helping, sharing and caring) and empathy towards others (the latter being evident only for girls).

Consistent with this pattern, research on social rejection or exclusion—typically defined as being actively ignored or disregarded by others (Taket, 2009)—has shown that negative social experiences are associated with reduced prosociality (Gunther Moor et al., 2012; Lin et al., 2025; Pakaslahti et al., 2002; Twenge et al., 2007; Wang & Qi, 2023; Wölfer & Scheithauer, 2013). However, social exclusion does not simply represent the absence of feeling socially supported or included, but rather constitutes a distinct form of social experience involving qualitatively different interactional dynamics, which is not the focus of the present research.

While existing research literature provides valuable insights into children's social status within their group and prosociality, most of these studies assessed social status based on ratings by teachers or peers (i.e. asking them to rate their classmates' respective perceived popularity or likability; for a detailed discussion on the definition and measurements, see Cillessen et al., 2011). Similarly, prosociality has often been assessed using general or self‐reported tendencies. In a recent study among fourth‐ and sixth‐graders, Sabato and Kogut (2021) challenged this positive association, by adding specific characteristics of the help recipient to the equation (namely, whether they are an ingroup or out‐group member; identified or unidentified). In that study, social status was gauged by asking children to nominate their actual social interactions with each peer. The children then engaged in an actual sharing task, in which they were asked to decide whether or not to share chocolate coins they had received for taking part in the study with another child from their class (vs. from another class). The findings revealed an intriguing pattern: higher‐status children exhibited a stronger tendency to share—but only when the recipient was an ingroup peer; when the recipient was an out‐group member, there was no difference between lower‐ and higher‐status children in their sharing behaviour. In similar vein, Asscheman et al. (2020) examined the link between the likability of children aged 8–12 years old and their sharing behaviour in a Dictator Game, as a function of their relationship with the help recipient (an anonymous child, a best friend, or a disliked peer). They found that less‐popular children shared less than others, irrespective of the recipient, while children who were liked by classmates shared more with close others, such as best friends. Together, these findings highlight the central role of social context in shaping children's sharing behaviour, suggesting that the association between social standing and prosociality may depend on whom the behaviour is directed towards.

In this context, it is important to note that most of this research has focused on prosocial behaviour directed towards a specific individual, often using paradigms such as Dictator or resource‐allocation games. In these settings, sharing decisions are made with respect to a particular recipient and may be shaped by interpersonal considerations such as egalitarian preferences, reciprocity, or the unique characteristics of the recipient (Falk & Fischbacher, 2006; Fehr et al., 2008). Accordingly, less is known about the link between children's social standing within a group and prosocial behaviour directed towards a group as a whole, rather than towards a specific individual.

Sharing with a group involves contributing resources to a collective pool that benefits multiple members simultaneously, typically examined in public goods paradigms. Decisions in such contexts may therefore rely on partially distinct motivations, including sensitivity to group norms, perceived responsibility towards the collective, and concerns about maintaining cooperation within the group (Keil et al., 2019; Vogelsang et al., 2014).

This distinction is particularly important in the context of children's peer relationships in school. In classroom settings, social interactions are closely tied to children's sense of belonging within the group. For example, more frequent and positive interactions with classmates strengthen feelings of belonging (Juvonen, 2006), whereas infrequent or negative interactions are associated with a reduced sense of belonging within the peer group (Mouratidis & Sideridis, 2009). Consequently, when examining prosocial behaviour as a function of children's social experiences, it is important to consider not only behaviour directed towards individual peers, but also behaviour directed towards the group as a collective. Building on this distinction, the present research focused on the relation between children's social inclusion within their peer group and their prosocial behaviour in a group‐based context. To that end, we used the *Public Goods Game* (PGG) to measure children's *actual behavioural* inclinations towards their group, rather than their general generosity as reported by significant others (i.e. teachers, parents, peers).

The Public Goods Game (hereafter PGG)—a widely used and well‐established method—designed to study prosocial behaviour in group settings. Specifically, it focuses on cooperative behaviour, since it presents participants with the social dilemma of choosing between their group's benefit, and their own (Ledyard, 1990). In this game, participants receive resources that they can either keep for themselves or contribute to the common pool. Individual contributions to the common pool are then collected and distributed equally among all group members. Thus, a cooperative strategy in this game is represented in one's decision to donate all of one's resources to the common pool, resulting in the maximum payoff for the group. Conversely, a selfish strategy is to keep one's resources, while benefiting from the contribution of others to the common good.

Research among adults reveals that approximately 35%–60% of participants opt for the cooperative option and allocate resources to the group (at least in the first round), despite the temptation to free‐ride on others' contribution (e.g. Goeschl et al., 2020). This tendency, however, can change in a non‐cooperative environment (i.e. *conditional cooperation*—Fischbacher et al., 2001). Several studies have used the PGG among children of various demographics, and found that overall, children's contributions in the PGG broadly align with those observed in adults (e.g. Keil et al., 2017, 2019). Children, like adults, are also sensitive to non‐cooperative behaviour of peers, as early as the age of five (Corbit et al., 2023; Keil et al., 2017; Vogelsang et al., 2014). This finding is in line with those on children's sharing in other economic games (such as the *Dictator Game*, or the *Ultimatum* game—Fehr et al., 2008; Kogut, 2012)—indicating children's growing sensitivity to various features of sharing situations as they grow older (such as self‐representation considerations—Heyman et al., 2014; effort and status—Schmidt et al., 2016; or the recipient's neediness—Sabato & Kogut, 2019, 2020). However, as previously noted, the relation between children's sharing for the common good and their social inclusion within the group remains understudied.

Although our primary focus when addressing this gap was on establishing the behavioural patterns of group‐based sharing, it is essential to consider the theoretical mechanisms that may support these associations. First, experiences of inclusion may enhance a sense of belonging and group identification, which in turn motivate contributions to collective outcomes (Baumeister & Leary, 1995; Williams, 2007). This process is supported by recent work on the role of group membership in children's sharing behaviour. For example, Lenz et al. (2021) showed that kindergarteners are more willing to share with friends than with non‐friends. Similarly, children aged 5–9 (but not younger) were found to favour in‐group over out‐group members in mini‐Dictator Games (Yu et al., 2016) and in the prisoner dilemma (Prétôt et al., 2024), suggesting that children's prosocial behaviour is shaped not only by their general social standing but also by the boundaries of their social groups. Second, repeated social interactions within the peer group may foster expectations of reciprocity and the internalization of cooperative norms (House et al., 2019). Third, children's level of social inclusion may be associated with a greater sense of responsibility towards the collective. More socially included or centrally positioned children may feel a stronger obligation to contribute to collective outcomes (Willer, 2009). In group contexts such as public goods situations, this heightened sense of responsibility may motivate greater contributions to the collective good. Finally, children's decisions to share may reflect strategic social behaviour aimed at maintaining or improving social inclusion (Chávez et al., 2022; Sabato et al., 2021). These processes may be particularly relevant in childhood, when peer group dynamics are especially salient.

Building on these underlying processes, the present research aimed to contribute to the literature in several ways. First, children's level of social inclusion within the group was assessed behaviourally, based on their actual interactions with peers, rather than on general ratings of popularity or likability. In Study 1, social inclusion was experimentally manipulated using a modified Cyberball paradigm that varied the degree of inclusion through the number of ball tosses received. In Study 2, social inclusion was assessed in a naturalistic setting based on children's peer interactions within their classroom.

Second, unlike prior work that has primarily focused on prosocial decisions towards individual recipients, the current studies examined children's willingness to contribute to a collective outcome, as operationalized through the PGG, in which individual contributions benefit the group as a whole. Importantly, this approach captures actual behavioural contributions rather than self‐reported tendencies, while also allowing for the possibility of free riding, whereby individuals may benefit from others' contributions without contributing themselves.

Finally, whereas research in adult populations has extensively examined the links between social standing, group processes and prosocial behaviour (Kafashan et al., 2014; see Piff & Robinson, 2017 for a review), relatively little is known about these dynamics in childhood, particularly using behavioural measures and group‐based paradigms. Examining these processes in children may therefore provide important insights into the developmental origins of cooperation within peer groups. The present research addresses these gaps by examining how social inclusion relates to children's group‐based cooperation.

## THE PRESENT STUDY

The present study sought to examine how levels of social inclusion within a group relate to children's sharing for the common good across two contexts: situationally induced inclusion in an experimental setting (Study 1) and more stable inclusion within natural peer group—the classroom (Study 2).

In Study 1, levels of social inclusion within a group were experimentally manipulated using a modified version of the Cyberball paradigm (Williams & Jarvis, 2006)—a virtual ball‐tossing game that allows precise control over the number of throws participants receive. While Cyberball is often used to create a binary experience of inclusion versus exclusion, we employed a five‐level manipulation of social inclusion, by systematically varying the number of ball tosses received. This approach builds on previous studies that have employed Cyberball with multiple conditions (e.g. Kawamoto et al., 2012; Van Bommel et al., 2016). It was designed to capture graded differences in children's experienced level of inclusion, reflecting the reality that children's social experiences in everyday peer interactions are rarely characterized by extreme exclusion or total inclusion, but rather by continuous variation in social engagement. After the game, participants took part in an online PGG, in which they decided how many coins (if any) to contribute to the group with whom they had played Cyberball.

While in Study 1 levels of social inclusion were experimentally manipulated as graded variations in children's momentary experiences of inclusion by peers in the game, Study 2 aimed to extend this examination by assessing more stable, ongoing social interactions within their natural peer group in the classroom. Importantly, in the Israeli education system, children usually remain with the same children in the same class from first through sixth grade. This provides a central and enduring social context where peer group dynamics are shaped by long‐term, cumulative experiences, making the role of social inclusion within these groups particularly meaningful. Study 2 therefore enhances ecological validity by examining whether the effects observed in a controlled experimental setting generalize to real‐life social interactions within stable peer groups.

We focused on children aged 10–12 years—a developmental stage characterized by increasingly stable friendships and more firmly established social status within the peer group (Poulin & Chan, 2010). During this period, peer relationships become more influential, and children show greater awareness of their social environment (Jones & Rutland, 2020; Selman, 2007). This age is also marked by heightened sensitivity to social rejection (Pharo et al., 2011; Sebastian et al., 2010; Yan et al., 2024), coupled with a stronger tendency to adhere to social norms, such as sharing (Kogut, 2012).

Based on prior research linking social inclusion within the group to prosocial behaviour—particularly towards in‐group members (Over, 2018; Sabato & Kogut, 2021)—and evidence that frequent and positive social interactions foster belonging, internalization of prosocial norms and motivation to maintain or enhance peer relationships (Chávez et al., 2022; Gunther Moor et al., 2012; Juvonen, 2006; Wilson et al., 2014; Wölfer & Scheithauer, 2013), we hypothesized that children experiencing higher levels of social inclusion would share more with their group Specifically, levels of social inclusion within the group (manipulated by the number of throws received in the modified Cyberball game in Study 1, and operationalized through children's natural peer interactions in Study 2) would predict greater contributions to the common good in the PGG.

While the primary scope of this research focuses on the varying levels of social inclusion, we also conducted exploratory analyses of the zero‐throws condition. Given that social exclusion may not necessarily reflect the lower end of the inclusion continuum, we do not advance formal predictions regarding this condition. Instead, we examined whether the zero‐toss condition aligns with the overall linear trend observed across levels of inclusion.

Both studies obtained ethical clearance from the authors' department Ethics Committees and the Ministry of Education. Preregistration of Study 1 is available at https://aspredicted.org/5hj2p8.pdf. Data for both studies are available at https://osf.io/ezwjs/?view_only=f7ac50a6febd49b39b8795efc493daeb.

## STUDY 1

### Participants

A total of 165 children, aged 10–12 years old (49% girls; *M*_age_ = 11.26, *SD* = 0.66) from all areas of Israel were recruited through an online research panel. Participation was limited to Hebrew‐speaking children from non‐orthodox households. Based on parental reports, participants predominantly came from average socioeconomic status (67.9%), with smaller proportions reporting above‐average (21.2%), below‐average (8.5%), and extreme categories (much above‐average: 2.4%; much below‐average: 1.8%). Most parents reported higher education, including 40% with a bachelor's degree, 30% with a master's degree, and 5% with a doctoral degree, while 15% reported post‐secondary vocational training and 10% reported high school education. Overall, the sample represents a WEIRD (Western, Educated, Industrialized, Rich, Democratic) population. Based on the size of the available pool of children within the required age range in the panel, we sought to recruit approximately 150 participants. A sensitivity analysis using G‐Power (Faul et al., 2007) for a regression model with one predictor (group assignment), assuming 80% power and a significance level of *α* = .05, indicated that the study can detect a small effect size of *f* ^2^ = 0.05. This corresponds to the ability to identify effects explaining approximately 5% of the variance in the outcome variable.

### Procedure and materials

The panel first contacted parents for their informed consent, and asked them to complete a short initial questionnaire, that included demographic information and an assessment of their child's ability to participate independently in the study. Participants then completed the study in a single session, beginning with providing informed consent a declaration that they were able to complete the study independently. The study consisted of two stages: a modified Cyberball game (Williams & Jarvis, 2006) designed to manipulate participants' feelings of social inclusion within the group, followed by a PGG to assess sharing behaviour.

#### The modified Cyberball game (Williams & Jarvis, 2006)

Children's level of social inclusion was experimentally manipulated as a function of the number of tosses received in a modified Cyberball paradigm. To that end, participants were randomly assigned to one of five experimental groups: receiving 0, 2, 4, 6 or 8 tosses out of a total of 30 tosses. Then, as a manipulation check, participants rated (1) how often the ball was thrown to them (on a 5‐point scale ranging from *Not at all* to *Many times*) and (2) the extent to which they felt part of the group (on a 5‐point scale, from *Not at all* to *Very much*). Children were told that the other players were from different schools and were playing on their own computers.

#### The PGG (adapted from Marwell & Ames, 1980)

The PGG consisted of two rounds, in which participants were provided with five coins online and could decide how many to contribute to a group pot—with pooled contributions subsequently distributed equally among all group members. The ‘group’ corresponded to the peers they had played with in the Cyberball game. The PGG consisted of two rounds^1^ to give participants the opportunity to adjust their behaviour based on their experience in the first round, while maintaining comparability across children. Using two rounds also allowed for a slightly more reliable measure of cooperative tendencies without overburdening the participants. In each round, participants first indicated whether they wished to contribute from their prizes (*Yes* or *No*), and if they chose *Yes*, the number of coins they were willing to contribute (1–5). The second round was presented as a new round, with the public pot reset to zero. The game started with the following explanation, which was also illustrated with images (See Supporting Information):

‘The next game is one in which you can earn coins, that can be exchanged for prizes at the end of the game. You will play with the same group you played with in the tossing game. In addition to giving coins to each child, we also give coins to the group pot—this is a pot for the whole group. At the end of the game, it will be divided among all the children in the group. Right now, the group pot is empty, so we ask the children in the group if they are willing to contribute some of their own coins to the group pot. You will now participate in two rounds of coin distribution, in which you can choose how many coins to keep for yourself and how many to put in the group pot. In each round, the amount you put in the group pot will be doubled by us!’

Next, participants took part in another short ball‐toss game, in which they received the maximum number of tosses, to enhance positive feelings and to mitigate any potential negative effects of the previous experimental manipulation. At the end of the experiment, they were debriefed as follows:

‘Thank you for participating in this study! In this research, we examine whether the number of tosses children receive influences the number of coins they subsequently choose to share with the group. The characters you played with were not real children; they were computer‐generated avatars, and the game was entirely virtual. All participating children received a monetary reward instead of the coins. All children received the same amount, regardless of the number of coins they chose to keep in the game. The payment was transferred directly to your parent’.^2^

This procedure was designed based on ethical considerations, to preserve the privacy of the children's decisions and to minimize any potential discomfort or negative feelings that might arise from perceived unequal outcomes or from the Cyberball paradigm.

## RESULTS

### Manipulation check

To assess the effectiveness of the Cyberball manipulation, we computed a Pearson correlation between the condition (i.e. number of throws received) and the Manipulation Check Index—i.e. the mean of participants' ratings of perceived ball reception and perceived group inclusion. The correlation was strong and significant: *r* = .77, *p* < .001.

### Social inclusion within the group and sharing behaviour

Participants' contributions to the common good (averaged across the two rounds) ranged from 0 to 5 coins. In total, 15 participants contributed nothing, while 9 participants contributed the maximum of 5 coins. In the first round, 23 children contributed no coins, and 14 contributed 5 coins; in the second round, 20 contributed none and 16 contributed 5.

As a preliminary check, we examined whether children's age or gender influenced contributions in the PGG. A linear regression predicting mean contributions from age and gender was not significant, *F* (2, 161) = 0.49, *p* = .614, *R* ^2^ = .01, indicating that neither age (*b* = .14, *p* = .378) nor gender (*b* = .09, *p* = .652) had a detectable effect on children's sharing in the PGG.

A linear regression model predicting mean‐PGG contributions from the level of social inclusion in the Cyberball game was significant: *F* (1, 163) = 5.30, *p* = .023, *R* ^2^ = .03. As hypothesized, higher levels of social inclusion (i.e. higher number of throws received) significantly predicted greater sharing: *b* = .18, *t* (163) = 2.30, *p* = .023. We then tested this association for each round of the PGG separately. The results were significant for both rounds. In the first round, social inclusion level predicted sharing behaviour: *F* (1, 163) = 4.13, *p* = .044, *R* ^2^ = .02, *b* = .14, *t* (163) = 2.03, *p* = .044. The effect was similar in the second round: *F* (1, 163) = 6.59, *p* = .011, *R* ^2^ = .04, *b* = .20, *t* (163) = 2.56, *p* = .011. In both cases, higher inclusion levels were associated with a greater willingness to share resources for the common good.

### Exploratory analyses: The zero‐toss condition

As noted above, our primary focus was to examine how increasing levels of inclusion relate to children's sharing behaviour, rather than contrasting social exclusion with inclusion. Thus, the number of ball tosses was treated as a continuous behavioural index of social inclusion. However, the zero‐toss condition may differ in its qualitative features from lower levels of inclusion. Given the unique nature of this condition, we additionally conducted exploratory analyses focusing on whether children's sharing behaviour in the zero‐toss condition followed similar patterns to those observed across levels of inclusion.

First, a one‐way ANOVA comparing the five experimental conditions revealed no significant overall differences in mean contributions to the public good, *F* (4, 159) = 1.56, *p* = .189, *η* ^2^ = .038. Second, polynomial trend analyses indicated a significant linear trend across conditions, *t* (159) = 2.03, *p* = .023, with higher levels of social inclusion associated with greater contributions to the public good. Higher‐order trends (quadratic, cubic) were not significant.

Finally, planned contrasts were conducted to further characterize the pattern of results. A contrast comparing the zero‐toss condition to all other conditions was not significant, *t* (159) = −1.04, *p* = .298.

Overall, these exploratory analyses are consistent with a graded pattern of responding across levels of social inclusion, without clear evidence that the zero‐toss condition departs substantially from this trend. These exploratory comparisons should be interpreted cautiously, as the study was primarily designed to detect linear trends across levels of social inclusion rather than condition‐specific differences.

While Study 1 manipulated levels of social inclusion in a short‐term experimental setting, Study 2 extended this investigation by examining whether these patterns generalize to real‐world peer contexts, in which social relationships are more stable and embedded in ongoing interactions. Specifically, examining children's social inclusion within their classroom allowed us to test whether the observed effects extend to ecologically valid and meaningful peer environments that characterize children's everyday social experiences.

## STUDY 2

### Method

#### Participants

Participants were all fourth or fifth graders (age range: 10–11; *M*_age_ = 10.46, *SD* = 0.49) from three secular public elementary schools located in the central region of Israel. The schools served populations of an average socioeconomic status, according to the Ministry of Education ranking of state schools in the country (which are based on the socioeconomic background of the students, the per capita income of their respective families, the school's geographical location and the students' family country of origin). Overall, the sample represents a WEIRD population. All participating children were native Hebrew speakers and completed the questionnaire in that language. The data was gathered as part of a larger project that examined class climate. Only children whose parents had approved their participation took part in the study.

In line with the sample size targeted in Study 1, we sought to recruit a comparable number of participants for Study 2. Because this study included two measurement time points, we accounted for potential attrition by recruiting a larger initial sample. Accordingly, 299 children participated at Time 1 (51% girls; fourth graders: 165, fifth graders: 134). Of these, 249 children completed the study at both time points and were included in the final analyses (52% girls; fourth graders: 134, fifth graders: 115). Fifty children were unavailable at the second time point, due either to absence that day or participation in other school activities. A sensitivity analysis conducted using G*Power (Faul et al., 2007) indicated that this final sample size provides 80% power to detect a small effect (*f* ^2^ = .03) in a regression model with *α* = .05.

#### Procedure and materials

The study was conducted at two time points in a classroom setting by trained research assistants. At Timepoint 1, children completed the Social Inclusion measure. One week later, at Timepoint 2, they completed additional questionnaires as part of a broader project and participated in an online PGG, which each child played individually on a tablet. Data were collected at two time points to minimize any potential influence of the social inclusion measure on children's sharing behaviour.

At both time points, research assistants introduced themselves, explained that participation was voluntary, and emphasized that children could choose to opt out at any time (although all chose to participate). They then provided instructions for completing the relevant measures. To enable matching across the two sessions, participants identified themselves using their assigned number from the class list.

##### Social inclusion measure (from Sabato et al., 2021; Sabato & Kogut, 2021)

Levels of social inclusion were assessed using a sociometric measure based on the number of peer social interactions, as reported by children. The research assistant wrote down all the students' names along with their assigned serial numbers on the board, in the line with the class's student list. Each child then received a brief questionnaire, and told to write their assigned number (from the board) at the top of the questionnaire. Then, on a table format with columns labelled *Yes* and *No*, they were asked to indicate, for each classmate, whether they typically (1) engaged in play with that child during school breaks, (2) met them after school and (3) confided in them. Importantly, the research assistant assured the children that their responses would be confidential and anonymous, and that the final lists would not contain any names, and would remain undisclosed within their school community.

##### The PGG (adapted from Marwell & Ames, 1980)

The game followed the same procedure and instructions as in Study 1, but each of the two rounds included different sets of prizes (two types of candy).^3^ The task was conducted in a quiet room at the school, with small groups of children to ensure focus and minimize distractions. First, the research assistant introduced the game to the class, to make sure the children understood the rules (which were also presented in the text presented to them at the start of the task). She explained to the children that they would receive prizes during the game, and asked if they would like to contribute some of their prizes to a collective stock for the entire class. The research assistant emphasized that in accordance with the game's rules, the prizes contributed by each child would be doubled by the research assistants and evenly distributed among all participants at the conclusion of the game. They also stressed that there were no right or wrong decisions, and assured the children that their choices would remain anonymous. The game started with the following explanation, which was also illustrated with images (see Supporting Information):

‘All the children participating in our study receive prizes for their participation.

In addition to giving each child prizes, we also add prizes to the class pot—this is a pot for the entire class, which will be divided among all the children in the class at the end of the day. Right now, the class pot is empty. Therefore, we ask the children in the class if they are willing to contribute some of their own prizes to the class pot.

You will now take part in several rounds of prize distribution, where you can choose how many prizes to keep for yourself, and how many to put in the class pot. In each round, the amount you put in the class pot will be doubled by us! Please note: after each round, we will check how many prizes were collected in the class pot, and then empty it again for the next round (meaning that in each round, prize collection starts from zero).’

## RESULTS

Residuals of the Social Inclusion measure and the Sharing measure were checked for normality assumptions by inspecting Q–Q plots, skewness and kurtosis (within the limits of ±1). A Social Inclusion Index for each participant was computed, based on the number of children who had noted that they play with that child during school breaks, meet them after school, and confide in them (the average of the three categories). This measure ranged between 0.67 and 14.33, *M* = 6.69, *SD* = 2.53.

Participants' contributions to the common good (the average of the two rounds) ranged from 0 to 5—with 39 participants contributing nothing, and 42 who contributed 5 (in the first round: 51 contributed nothing, 50 contributed 5; in the second round: 53 contributed 0, 63 contributed 5). A linear regression was conducted, with social inclusion as the predictor and the mean number of prizes shared across both rounds as the outcome variable. The overall model was significant, *F* (1, 252) = 7.02, *p* = .009, *R* ^2^ = .03. As hypothesized, higher levels of social inclusion significantly predicted greater sharing, *b* = .16, *t* (252) = 2.65, *p* = .009. We then tested this association for each round of the PGG separately. The results were significant for both rounds. In the first round, social inclusion positively predicted sharing behaviour, *F* (1, 251) = 4.73, *p* = .030, *R* ^2^ = .02, *b* = .14, *t* (251) = 2.17, *p* = .030. The effect was similar in the second round, *F* (1, 247) = 7.85, *p* = .005, *R* ^2^ = .03, *b* = .17, *t* (247) = 2.80, *p* = .005. In both cases, higher levels of social inclusion were associated with a greater willingness to share resources for the common good.

## DISCUSSION

The present study sought to enhance our understanding of the association between levels of social inclusion and prosocial behaviour towards one's group during late childhood. Specifically, we focused on children's actual sharing with their group as a whole through the PGG, as a function of their social inclusion within the group—both within a temporary, experimentally created group of peers (Study 1), and within their natural school classroom groups (Study 2). Our findings indicated that, overall, children who experienced higher levels of inclusion in the modified Cyberball game, and those who experienced higher levels of social inclusion within their class, exhibited a greater inclination to share with their group at their own expense, compared with those of lower levels of social inclusion.

These findings are consistent with past research that found prosocial behaviour to be associated with individuals' feelings of social connectedness and perceived support (Angelis & Pensini, 2023; Lin et al., 2025; Pavey et al., 2011; Trombini & Kinias, 2022), and with developmental research showing that peer acceptance is linked to higher levels of prosocial tendencies (e.g. Kornbluh & Neal, 2016; LaFontana & Cillessen, 2002; Tur‐Porcar et al., 2018), including within classroom settings (Eggum‐Wilkens et al., 2014). Similar patterns have been observed in experimental studies that examined actual sharing behaviour in the Dictator Game as a function of children's social status in the classroom (Asscheman et al., 2020; Sabato & Kogut, 2021). Our study not only replicates this pattern, but also extends prior research by demonstrating this association and effect in terms of actual sharing for the common good, rather than sharing with a single Other. Specifically, the PGG presented each participant with the social dilemma of deciding between maximizing their respective resources (i.e. sharing nothing with the public pool, yet enjoying others' contributions) and maximizing the group's benefit (i.e. sharing to maximize the public pool, at one's own expense). While sharing with a specific individual is often driven by factors such as empathy, familiarity and expectations of reciprocity (Batson et al., 2015; Berg et al., 1995), sharing with a group involves distinct social‐cognitive processes. First, it highlights one's affiliation with the group and reinforces social identity as an ingroup member (Hogg, 2016). Second, it shifts the focus from responsibility towards a particular individual to a more diffuse sense of obligation towards the collective (Fehr & Gächter, 2000). Finally, because the recipient in group contexts is less clearly defined and more abstract, decisions may rely less on person‐specific information and more on generalized norms and expectations. These features are particularly relevant when considering children's level of inclusion within the group as a predictor of cooperative behaviour. Children with higher levels of social inclusion, who are more strongly identified with the group, are more likely to help ingroup members and to rely on group‐based norms even in the absence of a clearly defined recipient.

While the present studies demonstrated this behavioural pattern across both experimental and real‐life contexts, we did not directly assess the psychological mechanisms underlying this effect. Based on prior research, several complementary processes may account for these patterns. First, social inclusion may shape children's sense of belonging and group identification. Social experiences of inclusion are associated with more positive affect and a stronger sense of connection to the group (Juvonen, 2006; Lansu et al., 2021), which in turn may promote cooperative behaviour—a mechanism also implicated in classroom dynamics (Lansu, 2023; Wölfer & Scheithauer, 2013). Second, repeated engagement in social interactions within the group may foster the internalization of group norms and expectations of reciprocity within group members (House et al., 2019), leading to increased sharing with the group. In addition, such richer social experiences provide opportunities for social learning, thus leading to greater social emotional skills, and especially to stronger self‐regulation (Wilson et al., 2014). Contributing to the public good requires delaying immediate self‐interest—a process navigated more effectively by children with stronger self‐regulatory abilities (Glenn et al., 2021).

Third, children who are more socially included and embedded in the peer group may feel a stronger obligation to contribute to shared outcomes, consistent with social responsibility norms and expectations associated with higher‐status roles (Choukas‐Bradley et al., 2015). Moreover, prosocial behaviours may constitute a strategic social behaviour aimed at maintaining or enhancing peer relationships (Chávez et al., 2022; Sabato et al., 2021). In group contexts such as the classroom, where interactions are ongoing and interdependent, this sense of responsibility may motivate greater contributions to the public good.

Finally, children of higher social inclusion are more sensitive to social contexts (Wentzel et al., 2007). Prior research shows that socially included children are more responsive to cooperative (vs. competitive) situations, across both prosocial (Lansu & Cillessen, 2015) and negative behaviours (Lansu, 2023; Lansu et al., 2021). This suggests that children of higher social inclusion may strategically adjust their behaviour to align with situational expectations—which may account for their greater contribution to the public good in group settings. Future research should directly investigate these mechanisms, using measures such as affective responses, regulatory skills, or perceptions of group norms, to clarify how social experiences translate into cooperative behaviour.

Although the primary focus of the present research was on graded levels of social inclusion, we additionally examined the zero‐toss condition as an exploratory case of experimentally induced social exclusion. These analyses indicated that in the current paradigm, this condition did not deviate significantly from the overall linear trends observed across levels of inclusion. Prior research has shown that social exclusion can elicit negative affect and reduce prosociality (Gunther Moor et al., 2012; Twenge et al., 2007; Wölfer & Scheithauer, 2013), although some studies suggest more complex, compensatory responses where ostracized or rejected individuals may increase prosocial efforts to regain social standing (e.g. Smart Richman & Leary, 2009; Williams, 2007). It is important to note that the social inclusion measure in Study 2 focused on the frequency of positive social interactions within the group. Within this naturalistic sample, all children were nominated for at least some interactions; thus a zero‐nomination state was not observed in our ecological data. However, this measure was not designed to capture active peer rejection or ostracism (e.g. through negative nominations). Given that social exclusion represents a qualitatively distinct social experience, future research should directly examine experiences of social exclusion and their specific effects on cooperative behaviour.

Taken together, both studies highlight the central role of social inclusion within the group in shaping children's cooperative decisions. Whether experienced through momentarily inclusion (in the Cyberball paradigm, Study 1), or reflected in real‐life settings (i.e. social inclusion within the classroom, Study 2), children who feel that they are valued members of their group appear more motivated to act in ways that support the collective good.

The present findings have both theoretical and practical implications. Theoretically, they highlight how children's social experiences shape prosocial behaviour within a group context, showing that contributions to a collective good are influenced by both momentary and enduring peer interactions. This underscores the importance of considering both situational and stable social factors when modelling prosocial behaviour towards the group. Moreover, these findings also speak to the role of prosocial behaviour in shaping a more positive and secure classroom climate, as cooperative and inclusive actions among children may contribute to a greater sense of safety, belonging and mutual support within the peer group. Practically, the results suggest that fostering inclusive peer interactions and enhancing children's sense of belonging within the classroom can promote not only cooperative behaviour but also a more supportive and psychologically safe group environment.

Future research may further consider potential moderators of these effects, such as children's level of identification with the peer group, the broader classroom social climate, and individual differences in sensitivity to peer context. These moderators may help delineate boundary conditions of the observed effects and clarify for whom and under which conditions social inclusion is most strongly associated with prosocial behaviour towards the group.

### Study limitations

Although our study provides an initial examination of the role of social inclusion in children's actual sharing for the common good, it is not without limitations. One limitation concerns the design of the PGG across the two rounds. While some studies reveal participants' decision after the first round (Vogelsang et al., 2014), we chose not to provide this information in order to avoid influencing subsequent decisions (e.g. through conditional cooperation—Fischbacher et al., 2001). Although we observed consistent patterns across both rounds and in the average amount shared, future studies might directly examine how revealing peers' choices affects children's contributions, and whether this interacts with social inclusion. Building on previous studies that found greater strategic thinking among accepted children (e.g. in children's motivation to help—Sabato et al., 2021), we might expect such children to better adhere to group norms or the common good when making subsequent decisions.

We also did not directly measure the underlying mechanisms linking children's social inclusion and their cooperative behaviour. While we proposed several plausible explanations—such as heightened sensitivity to social contexts, stronger fairness considerations, greater self‐regulation and richer internalization of social norms—these remain unexplored in the present study. Future research might incorporate direct measures of these processes—for example, by assessing children's emotional responses, regulatory skills, perception of social norms, or strategic decision‐making in group contexts, to better understand how social inclusion translates into prosocial behaviour.

Moreover, Study 2 assessed children's social inclusion in a natural classroom setting, offering strong ecological validity. However, because inclusion was observed rather than experimentally manipulated, and cooperative behaviour was measured only 1 week later, causal conclusions about the impact of real‐life social inclusion on prosocial behaviour cannot be drawn. Future research might employ longer‐term longitudinal designs to clarify causal mechanisms and to examine the lasting effects of social inclusion on cooperative behaviour.

Finally, in both studies the sample represents WEIRD societies (Western, Educated, Industrialized, Rich, Democratic)—which may limit the generalization of the findings. Research on prosociality among children points to sociocultural variation (Blake et al., 2015; Callaghan & Corbit, 2018; Marshall et al., 2022). Future research could examine whether the observed associations between social inclusion and sharing with the group replicate in contexts with differing social norms around cooperation and sharing.

## CONCLUSIONS

Across two complementary studies, the present findings indicate that children's willingness to contribute to a shared group resource is closely tied to, and affected by, their experience of social inclusion. Whether reflected in stable peer relationships within the classroom or momentarily shaped through experimentally induced inclusion, feeling socially included was associated with greater cooperative sharing. By focusing on actual contributions to a public good, this work extends prior research on social inclusion and prosocial behaviour beyond helping a single recipient to group‐level cooperation. The findings highlight the role of social relationships and perceived standing within the group in shaping children's collective contributions.

## AUTHOR CONTRIBUTIONS

**Hagit Sabato:** Conceptualization; funding acquisition; methodology; writing – review and editing; supervision. **Yael Malin:** Conceptualization; investigation; writing – original draft; methodology; formal analysis; data curation.

## FUNDING INFORMATION

Israel Science Foundation (ISF) Grant #1830/22, awarded to H. Sabato.

## CONFLICT OF INTEREST STATEMENT

The authors declare that they have no conflicts of interest.

## Supporting information


Data S1.

## Data Availability

The data that support the findings of this study are openly available in OSF at https://osf.io/ezwjs/?view_only=f7ac50a6febd49b39b8795efc493daeb, reference number Ezwjs.

## Footnotes

^1^
Other studies employing the same game varied the number of rounds played—from one to nine rounds (e.g. Keil et al., 2019).

^2^
At the end of the study, parents were informed that their children had participated in an allocation task and were told they would receive their earnings. Accordingly, an additional monetary bonus (equivalent to the highest possible amount, to prevent disappointment and to preserve the privacy of the decisions) was provided to the parents in addition to the panel‐provided participation payment. Parents were asked to pass on this bonus to their children in the form of a small gift.

^3^
As is common in PGG studies with children, tokens were replaced with prizes, which—unlike in Study 1—was feasible in this setting (e.g. Keil et al., 2017, 2019; Vogelsang et al., 2014). At the end of the study, all children received an equal number of prizes (equivalent to the highest possible value), regardless of their decisions during the game, to prevent feelings of dissatisfaction and to preserve the privacy of their decisions.
