# Supplementary material for: Sharing for the common good: Children's levels of social inclusion within the group and their sharing decisions in a public goods game
Source: Br J Soc Psychol. 2026 Jun 4;65(3):e70105. doi: 10.1111/bjso.70105 (PMC13238313; doi:10.1111/bjso.70105)
Supplement: Supplementary file 1 — Data S1. [file BJSO-65-0-s001.docx]

**The Public Goods Game (Illustration)**

The illustrations shown here depict the instructions provided to participants in Study 1 and Study 2 prior to the Public Goods Game. Gender-specific illustrations were used, such that female and male participants viewed different versions aligned with their gender

**Study 1:**

You receive these 5 coins:


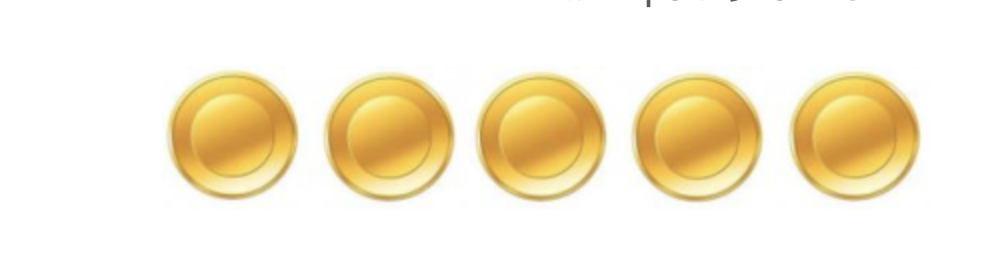


These coins belong to you, and you can choose to keep them all for yourself. Additionally, you have the option to contribute coins to the class shared basket. Here's how the contribution works:

- If you decide to contribute 1 coin, we will add 2 coins to the class basket.
- If you decide to contribute 2 coins, we will add 4 coins to the class basket.
- If you decide to contribute 3 coins, we will add 6 coins to the class basket.
- If you decide to contribute 4 coins, we will add 8 coins to the class basket.
- If you decide to contribute 5 coins, we will add 10 coins to the class basket.

For instance, if you choose to contribute 2 coins to the class basket, we will add 4 coins to the basket, leaving you with 3 coins, as illustrated in the image:

**
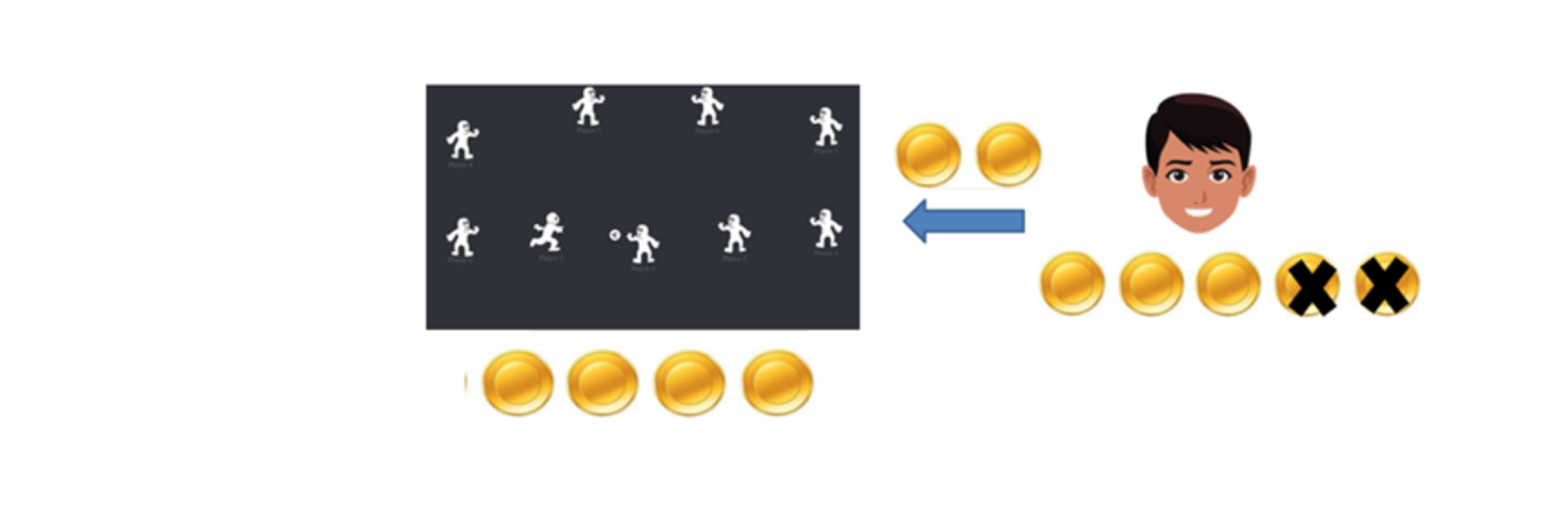

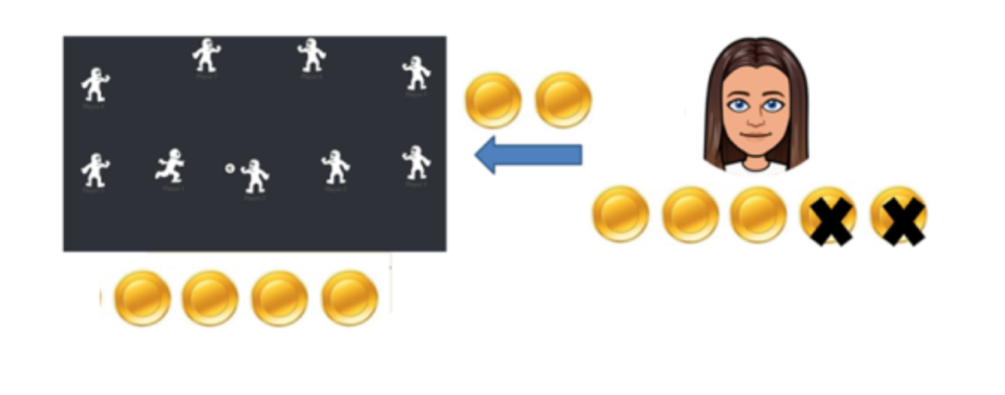
**

**Study 2:**

**
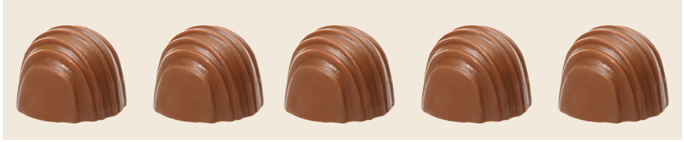
**You receive these 5 chocolates:

These chocolates belong to you, and you can choose to keep them all for yourself. Additionally, you have the option to contribute chocolates to the class shared basket. Here's how the contribution works:

- If you decide to contribute 1 chocolate, we will add 2 chocolates to the class basket.
- If you decide to contribute 2 chocolates, we will add 4 chocolates to the class basket.
- If you decide to contribute 3 chocolates, we will add 6 chocolates to the class basket.
- If you decide to contribute 4 chocolates, we will add 8 chocolates to the class basket.
- If you decide to contribute 5 chocolates, we will add 10 chocolates to the class basket.


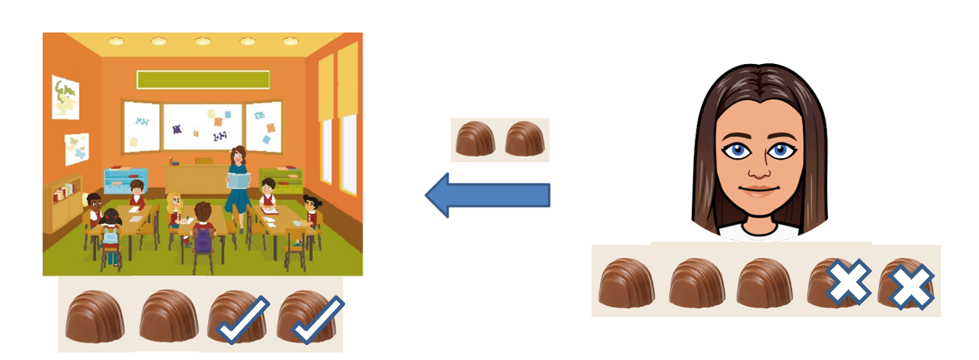

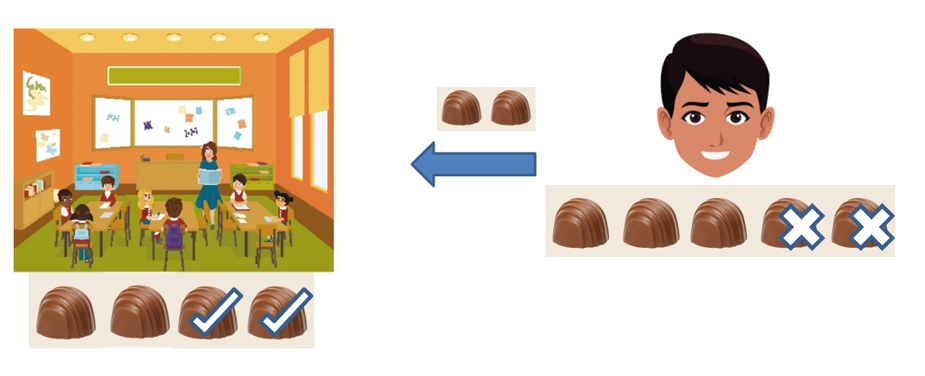
For instance, if you choose to contribute 2 chocolates to the class basket, we will add 4 chocolates to the basket, leaving you with 3 chocolates, as illustrated in the image:
